# Pre-diagnosis Dairy Product Intake and Ovarian Cancer Mortality: Results From the Ovarian Cancer Follow-Up Study (OOPS)

**DOI:** 10.3389/fnut.2021.750801

**Published:** 2021-10-29

**Authors:** Luo Jiang, Ting-Ting Gong, Song Gao, Xiu-Qin Li, Fang-Hua Liu, Zhao-Yan Wen, Yi-Fan Wei, Shi Yan, Rui Hou, Qi-Jun Wu

**Affiliations:** ^1^Department of Ultrasound, Shengjing Hospital of China Medical University, Shenyang, China; ^2^Department of Obstetrics and Gynecology, Shengjing Hospital of China Medical University, Shenyang, China; ^3^Department of Clinical Epidemiology, Shengjing Hospital of China Medical University, Shenyang, China; ^4^Clinical Research Center, Shengjing Hospital of China Medical University, Shenyang, China

**Keywords:** cohort, dairy, mortality, ovarian cancer, prognosis, survival

## Abstract

**Background:** Dairy product consumption is associated with ovarian cancer (OC) incidence. However, limited evidence is available on its influence on OC mortality.

**Methods:** The association between pre-diagnostic dairy product intake and OC mortality was investigated in the OC follow-up study, which included a hospital-based cohort (*n* = 853) of women diagnosed with epithelial OC between 2015 and 2020. Pre-diagnosis diet information was collected using a validated food frequency questionnaire. Deaths were ascertained up to March 31, 2021 via death registry linkage. Cox proportional hazards model was used to estimate the adjusted hazard ratio (HR) and 95% confidence interval (CI) for the aforementioned association.

**Results:** A total of 130 women died during the median follow-up of 37.2 months (interquartile: 24.7–50.2 months). Comparisons of highest to lowest tertile intake showed that pre-diagnosis dairy product use was associated with total OC mortality (HR = 2.03, 95% CI = 1.21–3.40, *p* trend = 0.06). In addition, short survival was separately associated with protein (HR = 2.09, 95% CI = 1.25–3.49, *p* trend < 0.05), fat (HR = 2.16, 95% CI = 1.30–3.61, *p* trend < 0.05), and calcium (HR = 2.03, 95% CI = 1.21–3.4, *p* trend = 0.06) from dairy intake. Similar positive magnitudes were observed for menopausal status, residual lesions, histological type, and body mass index, although not all of these factors showed statistical significance.

**Conclusion:** Pre-diagnosis dairy product consumption, including protein, fat, and calcium from dairy intake, was associated with higher mortality among OC survivors.

## Introduction

Ovarian cancer (OC) is one of the most fatal gynecological malignancies, with an estimated 313,959 new cases and 207,252 new deaths globally in 2020 (1). In China, OC is the second leading cause of gynecological malignancy death, with ~25,000 new cases and 22,000 new deaths in 2015 (2). Since there are few early specific symptoms, a high proportion of women are diagnosed at advanced stages when the therapeutic effect is poor and the fatality rate is high (3). Although the 5-year survival rate has increased in recent years, it was still <50% in China (4), which seriously threatens women's health. Evidence suggests that several factors can influence the OC prognosis, including histotype, stage of disease at diagnosis, volume of residual disease after primary debulking surgery, parity, and number of ovulatory cycles (3, 5, 6). However, most of these factors are difficult to modify. In the last decade, increasing evidence has suggested that diet is a feasible intervention target, which might affect survival in OC patients (7–9).

Dairy products are an important and common part of daily diet. Dairy products are rich in protein, fat, and calcium. The fat in dairy products may be related to high levels of circulating estrogen and insulin-like growth factor-1, which may be associated with poor OC prognosis (10–14). Several studies have investigated the relationship between pre-diagnosis consumption of milk or dairy products and OC prognosis (7–9, 15, 16). Among them, three studies have reported a null association (8, 9, 16). However, some investigations have generated different results. For example, Nagle et al. found a modest relationship between pre-diagnosis dairy intake and poor survival for 609 Australian OC patients (15). Only one prospective cohort study conducted in the U.S. has reported a significant negative association between all types of milk consumption and OC survival (7). This inconsistent evidence might be attributed to different study design, population, exposure assessment, and adjustment for potential confounders. Furthermore, none of these studies has further analyzed the association between main nutrients in pre-diagnosis dairy product consumption and OC mortality. To the best of our knowledge, no study has explored the effect of dairy products on the survival of Chinese women with OC who may have different daily intake and consume different types of dairy compared to the American and European population.

Therefore, a prospective follow-up study was conducted to investigate the association between pre-diagnosis consumption of dairy products and related nutrients, including protein, fat, and calcium, and OC prognosis in China.

## Materials and Methods

### Study Population

**Figure 1:**
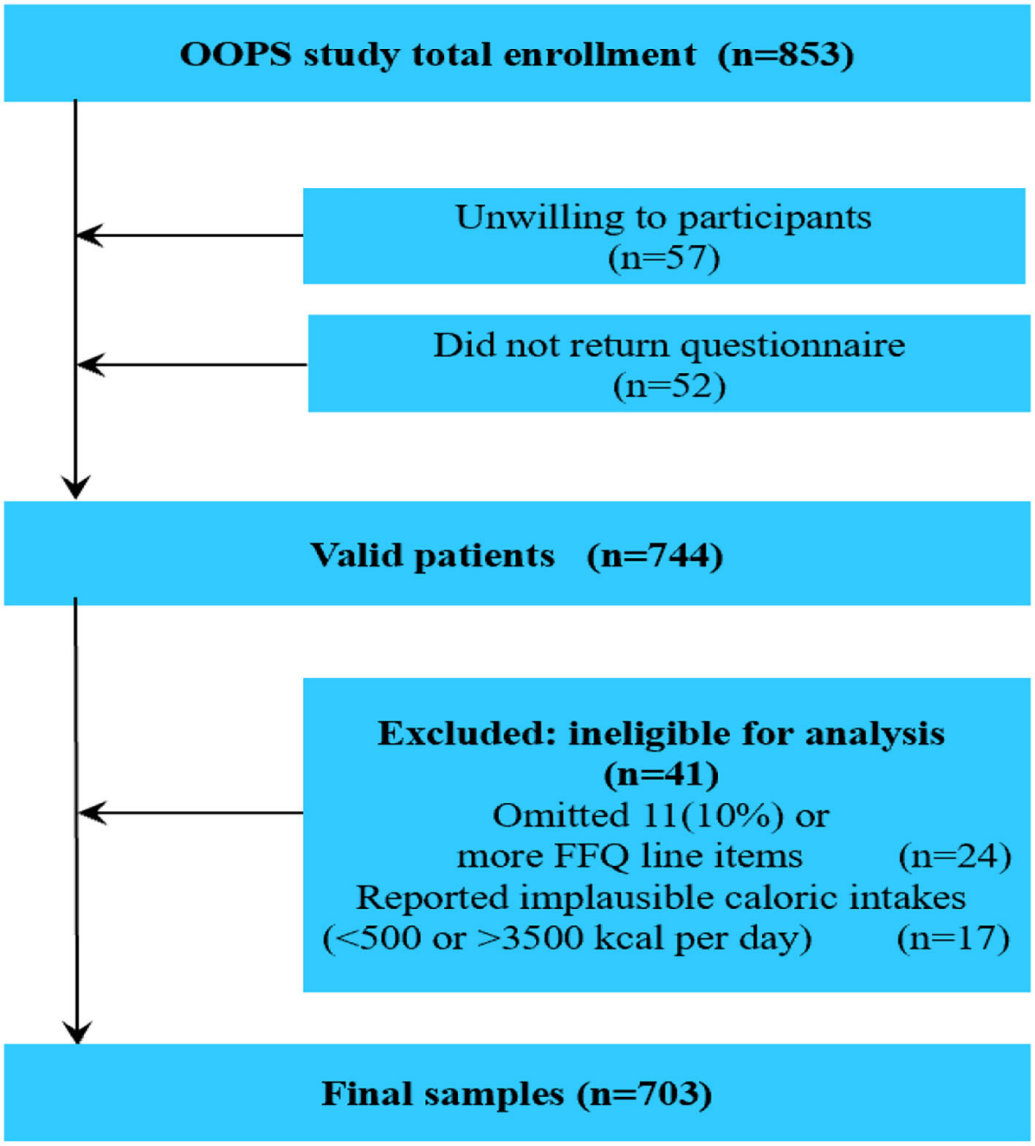
Flow of participants through study.

The OC follow-up study (OOPS) is a prospective longitudinal cohort study of newly diagnosed OC patients. Participants were recruited for the purpose of collecting demographic, clinical, and lifestyle data in order to assess their associations with cancer-related outcomes. The study was approved by the Institutional Review Board of the Ethics Committee of Shengjing Hospital of China Medical University. All women provided signed consent to participate. Based on traditional statistics and previous published studies, we set α = 0.05, Z_1−0.05/2_ = 1.96, Z_β_ = 1.28, P_0_ = 0.30, RR = 1.40, P_1_ = 0.42. And, we calculated the sample size is 662. Actually, a total of 853 women aged 18–79 years who were newly diagnosed with OC were identified between January 2015 and December 2020. Of these, 796 women agreed to participate and 744 (93%) returned the completed study questionnaire. After excluding participants who reported significantly abnormal caloric intake (<500 or > 3,500 calories per day; *n* = 17) or left 11 (10%) or more food items blank (*n* = 24), dietary data were available for 703 women with OC (Figure 1), which reached the statistical power.

### Data Collection

Information on demographic and lifestyle factors was collected in person using a self-administered questionnaire, which included information on diet, smoking status, alcohol intake status, tea intake status, menopausal status, parity, education, income, and amount of physical activity. Anthropometrics, including weight and height [used to calculate body mass index (BMI)], were measured at baseline. In addition, clinically relevant covariates included age at diagnosis, histological type, histopathologic grade, International Federation of Gynecology and Obstetrics (FIGO) stage, residual lesions, and comorbidities. Information on these covariates was collected from the electronic medical records of the Shengjing hospital information system.

### Dietary Exposure Assessment

Dietary intake was assessed at recruitment via a 111-item food frequency questionnaire (FFQ), which was an extended version of a previously validated FFQ (with an addition of 11 food items) used in the Tianjin Chronic Low-grade Systemic Inflammation and Health cohort study (17, 18). Participants were required to recall their accustomed intake of these food items during the year prior to OC diagnosis. Seven response categories were provided for each food item (i.e., almost never, less than once a week, once a week, two to three times a week, four to six times a week, once a day, and two or more times a day). Total dairy product intake was calculated by summing up intake amounts of whole milk, low-fat dairy, yogurt, and cheese. Intakes of different dairy products in grams/day were computed by multiplying consumption frequencies per day and fitted portion sizes (g/time). In addition, consumption of protein, fat, and calcium was computed from the above dairy products. Nutrient intake was determined by multiplying the frequency of consumption of each food by the nutrient content of the specified portions. Nutrient intake was estimated based on the Chinese Food Composition Tables (19).

### Follow-Up and Outcome

Information on the vital status of participants was determined using data extracted from the medical records every 6 months and by active follow-up. All-cause mortality was the endpoint for follow-up. Survival time was defined as the interval between histologic diagnosis and date of death from any cause or the date of last follow-up (March 31, 2021) for patients who were still alive.

### Statistical Analysis

Differences in general and clinical characteristics across dairy product intake categories were assessed using one-way ANOVA or the Kruskal–Wallis test for continuous variables, and the χ^2^ test for categorical variables. The Kaplan–Meier technique was used to plot crude survival curves and estimate crude overall survival probabilities. Cox proportional hazards regression was used to calculate the hazard ratio (HR) and 95% confidence interval (CI) for the association of baseline dairy products and relative nutrient intake with overall survival. The proportional hazards assumption was evaluated by including an interaction term between each activity variable and log survival time. No violations were observed (all *p* > 0.05). Dairy product intake was categorized by tertile distribution, where the lowest tertile served as the reference group. Tests for linear trends were carried out by assigning the median value of consumption for each tertile of dairy products and relative nutrients and treating it as a continuous variable in the respective regression model. To control for confounding factors, the model was adjusted for age at diagnosis (<50, ≥50 years), total energy intake (continuous, kcal), BMI (continuous, kg/m^2^), comorbidities (yes or no), diet change (yes or no), dietary pattern (derived using principal components for factor analysis), education (junior secondary or below, senior high school/technical secondary school, and junior college/university or above), FIGO stage (I–II, III–IV, and unknown), histological type (serous, non-serous), histopathologic grade (well, moderate, and poorly differentiated), menopausal status (yes or no), parity (≤ 1, ≥2), physical activity (continuous), residual lesions (none, <1, ≥1 cm), and smoking status (yes or no). Selection of covariates for the final model was based on clinical significance, previous studies, and degree of correlation with the exposure.

Stratified exploratory analyses were also performed using categories of menopausal status (“no” compared to “yes”), residual lesions (“no” compared to “yes”), histological type (serous compared to non-serous), and BMI (<25 compared to ≥25 kg/m^2^). Respective multiplicative interaction terms in the multivariable-adjusted models were tested by including the cross product of the dairy products or relative nutrients as a continuous variable and the potential effect modifier as a continuous or categorical variable, as appropriate. In addition, the association between pre-diagnosis dairy product intake and overall survival in stage III or IV OC patients was analyzed. All analyses were performed using SAS version 9.4 (SAS Institute, Cary, NC, USA). Two-sided *P*-values of < 0.05 were considered statistically significant.

## Results

General characteristics of 703 OC patients organized by tertiles of total dairy consumption are listed in Table 1. Patients with a higher total dairy product intake were more likely to consume total energy, meat, eggs, fish and seafood, beans and bean products, vegetables, and fruits, and had less parity. No differences in other listed variables were observed. Among the 703 OC patients included in the analysis, 130 deaths occurred during a median follow-up of 37.17 months (interquartile: 24.73–50.17 months). Non-serous histological subtype, later-stage disease, and greater residual disease were statistically significantly associated with worse survival in this cohort (Table 2).

**Table 1:** General characteristics of ovarian cancer patients according to dairy products (*N* = 703).

**Variables**	**Total dairy products consumption (g/day)**			***P*** **-value**
	**T1 (<17.46)**	**T2 (17.46–90.00)**	**T3 (≥90.00)**	
No. of patients	232	235	237	
Age at diagnosis (years), Median (IQR)	53.00 (48.00–60.00)	53.50 (47.00–60.00)	53.00 (48.00–61.00)	0.56
Follow–up time (m), Median (IQR)	33.83 (23.25–47.30)	30.92 (19.77–47.87)	29.60 (17.63–45.23)	0.23
Body mass index (kg/m^2^), Median (IQR)	23.30 (21.30–25.25)	23.30 (20.70–25.00)	23.30 (20.80–25.00)	0.70
Physical activity (MET/hours/days), Median (IQR)	14.15 (6.15–21.55)	14.10 (7.30–22.00)	13.60 (6.10–22.90)	0.85
Diet intake (Mean ± SD)				
Total energy (kcal/d)	1,168.06 ± 394.29	1,406.47 ± 472.09	1,786.04 ± 585.62	<0.05
Meat (g/day)	27.76 ± 22.76	36.70 ± 28.23	44.49 ± 33.91	<0.05
Eggs (g/day)	31.83 ± 26.23	34.71 ± 26.34	46.56 ± 26.65	<0.05
Fish and seafood (g/day)	22.60 ± 31.63	27.43± 24.66	35.39 ± 32.70	<0.05
Beans and bean products (g/day)	68.45 ± 68.97	85.45 ± 77.06	101.55 ± 85.11	<0.05
Vegetables (g/day)	192.81 ± 122.42	202.72 ± 108.51	246.53 ± 127.06	<0.05
Fruits (g/day)	153.73 ± 132.59	189.81 ± 152.41	239.45 ± 173.92	<0.05
Diet change (*n*, %)				0.07
No	188 (81.03)	169 (72.22)	178 (75.11)	
Yes	44 (18.97)	69 (27.78)	59 (24.89)	
Smoke status (*n*, %)				0.15
No	203 (87.50)	212 (90.60)	220 (92.83)	
Yes	29 (12.50)	22 (9.40)	17 (7.17)	
Alcohol intake (*n*, %)				0.08
No	194 (83.62)	181 (77.35)	179 (75.53)	
Yes	38 (16.38)	53 (22.65)	58 (24.47)	
Tea drinking (*n*, %)				0.60
No	161 (69.40)	161 (68.80)	155 (65.40)	
Yes	71 (30.60)	73 (31.20)	82 (34.60)	
Menopausal status (*n*, %)				0.48
No	58 (25.0)	66 (28.21)	71 (29.96)	
Yes	174 (75.0)	168 (71.79)	166 (70.04)	
Parity (*n*, %)				<0.05
≤ 1	144 (62.07)	172 (73.50)	189 (79.75)	
≥2	88 (37.93)	62 (26.50)	48 (20.25)	
Educational level (*n*, %)				0.09
Junior secondary or below	140 (60.34)	112 (47.86)	123 (51.90)	
Senior high school/technical secondary school	42 (18.11)	52 (22.22)	53 (22.36)	
Junior college/university or above	50 (21.55)	70 (29.92)	61 (25.74)	
Income per month (Yuan), (*n*, %)				0.44
<5,000	145 (62.50)	144 (61.54)	132 (55.70)	
5,000 to <10,000	59 (25.43)	59 (25.21)	76 (32.07)	
≥10,000	28 (12.07)	31 (13.25)	29 (12.23)	

*IQR, interquartile range; MET, metabolic equivalent task; SD, standard deviation; T, tertile*.

**Table 2:** Selected clinical characteristics and associations with all-cause mortality among women diagnosed with ovarian cancer (*N* = 703).

**Characteristic**	**No. of deaths/total (%)**	**Crude HR** **(95% CI)**	**Adjusted HR^a^ (95% CI)**
**Age at diagnosis**			
≤ 50	45/258 (17.44)	1.00 (ref)	1.00 (ref)
>50	85/445 (19.10)	1.18 (0.82–1.70)	1.24 (0.85–1.79)
**Histological type**			
Serous	92/479 (19.21)	1.00 (ref)	1.00 (ref)
Non-serous	38/224 (16.96)	0.87 (0.59–1.27)	1.71 (1.11–2.66)
**Histopathologic grade**			
Well-differentiated	5/56 (8.93)	1.00 (ref)	1.00 (ref)
Moderately differentiated	7/48 (14.58)	1.44 (0.46–4.57)	1.12 (0.35–3.57)
Poorly differentiated	118/599 (19.70)	2.32 (0.95–5.67)	1.76 (0.70–4.43)
**FIGO stage**			
I–II	41/342 (11.99)	1.00 (ref)	1.00 (ref)
III–IV	89/338 (26.33)	2.75 (1.89–4.00)	2.54 (1.65–3.91)
**Residual lesions**			
No	82/553 (14.83)	1.00 (ref)	1.00 (ref)
<1 cm	31/106 (29.25)	2.22 (1.47–3.36)	1.73 (1.11–2.68)
≥1 cm	17/44 (38.64)	3.18 (1.89–5.37)	2.41 (1.39–4.16)
**Comorbidities**			
No	74/393 (18.83)	1.00 (ref)	1.00 (ref)
Yes	56/310 (18.06)	0.82 (0.58–1.16)	0.97 (0.68–1.38)

a *Mutually adjusted for all other variables listed in the table*.

*CI, confidence interval; HR, hazard ratio; Ref, reference*.

**Figure 2:**
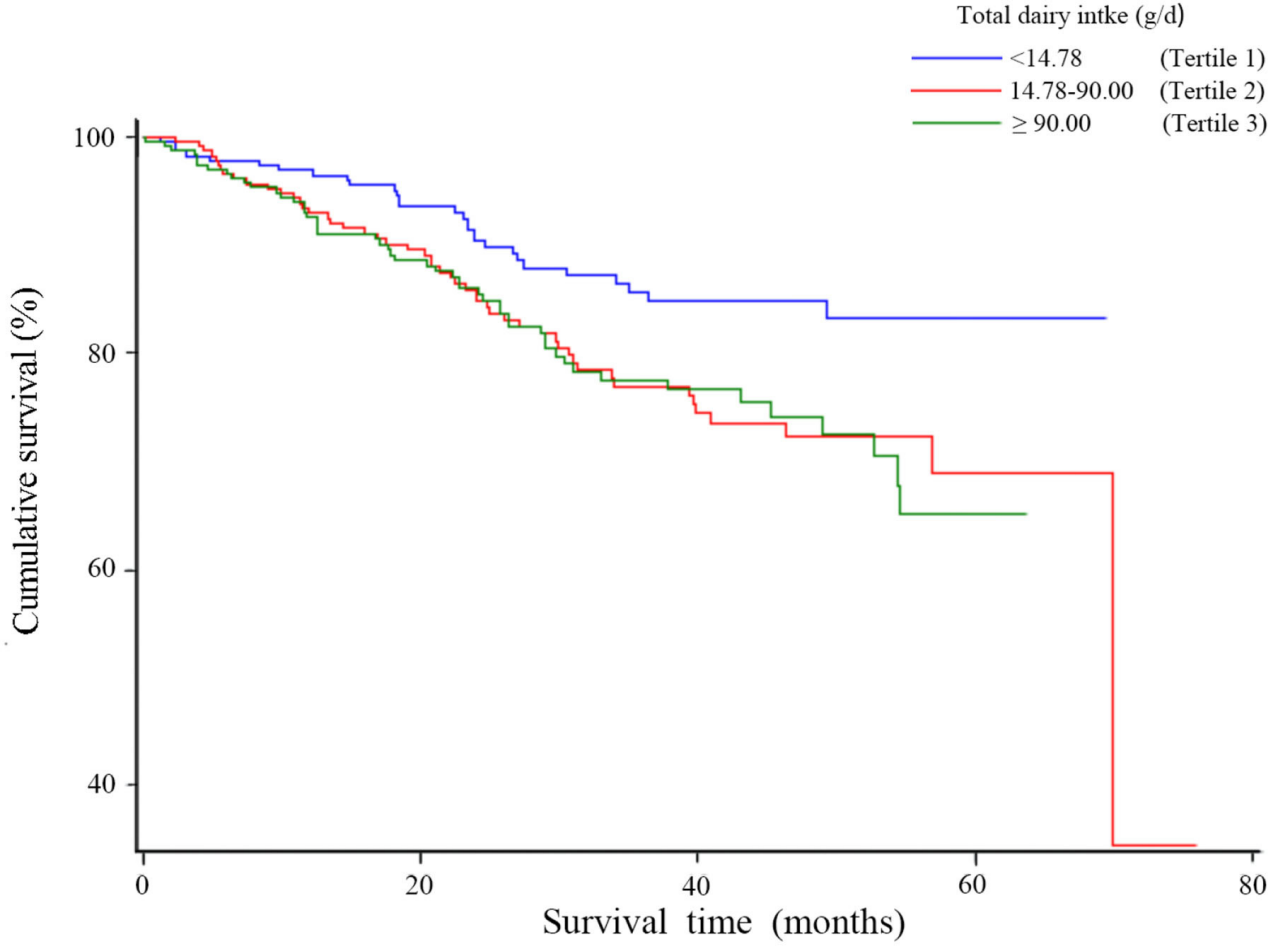
Kaplan-Meier survival curves for total dairy productions consumption.

Table 3 represents the associations between total dairy and relative nutrient intake and overall survival of OC. Patients with total dairy product intake in the highest tertile had worse overall survival compared to those in the lowest tertile (HR = 2.03, 95% CI = 1.21–3.40), though a linear trend was not evident (*p* trend = 0.06). A similar pattern was observed for calcium from dairy intake (HR_T3VS.T1_ = 2.03, 95% CI = 1.21–3.40, *p* trend = 0.06). Moreover, worse survival was evident for the highest tertile compared to the lowest tertile of protein from dairy intake (HR = 2.09, 95% CI = 1.25–3.49, *p* trend < 0.05) and for the highest tertile compared to the lowest tertile of fat from dairy intake (HR = 2.16, 95% CI = 1.30–3.61, *p* trend < 0.05). Figure 2 represents the association between total dairy intake and overall survival for OC. Compared to the lowest tertile of total dairy intake, survival was lower in patients in the highest intake tertile. Similar results for protein, fat, and calcium from dairy were observed (Supplementary Figures 1–3).

**Table 3:** Hazard ratio (95% CI) for overall survival among ovarian cancer patients according to total dairy and relative nutrients intake.

**Dietary variables**	**T1**	**T2**	**T3**	**P for trend^d^**
**Total dairy (g/day)**				
Rang of intake	<17.46	17.46–90.00	≥90.00	
Deaths, *N* (% of total deaths)	29 (22.31)	51 (39.23)	50 (38.46)	
Model 1^*a*^ (95% CI)	1.00 (ref)	1.78 (1.13–2.81)	1.84 (1.16–2.91)	<0.05
Model 2^*b*^ HR (95% CI)	1.00 (ref)	1.85 (1.16–2.95)	2.04 (1.23–3.39)	<0.05
Model 3^*c*^ HR (95% CI)	1.00 (ref)	2.00 (1.24–3.22)	2.03 (1.21–3.40)	0.06
**Protein from dairy (g/day)**				
Rang of intake	<0.49	0.49–3.00	≥3.00	
Deaths, *N* (% of total deaths)	29 (22.31)	51 (39.23)	50 (38.46)	
Model 1^*a*^ (95% CI)	1.00 (ref)	1.76 (1.11–2.78)	1.86 (1.18–2.94)	<0.05
Model 2^*b*^ HR (95% CI)	1.00 (ref)	1.83 (1.15–2.92)	2.07 (1.25–3.45)	<0.05
Model 3^*c*^ HR (95% CI)	1.00 (ref)	1.97 (1.22–3.17)	2.09 (1.25–3.49)	<0.05
**Fat from dairy (g/day)**				
Rang of intake	<0.39	0.39–2.78	≥2.78	
Deaths, *N* (% of total deaths)	29 (22.31)	49 (37.69)	52 (40.00)	
Model 1^*a*^ (95% CI)	1.00 (ref)	1.73 (1.09–2.74)	1.93 (1.22–3.04)	<0.05
Model 2^*b*^ HR (95% CI)	1.00 (ref)	1.81 (1.14–2.9)	2.18 (1.31–3.61)	<0.05
Model 3^*c*^ HR (95% CI)	1.00 (ref)	1.94 (1.20–3.13)	2.16 (1.30–3.61)	<0.05
**Calcium from dairy (mg/day)**				
Rang of intake	<15.79	15.79–100.89	≥100.89	
Deaths, N (% of total deaths)	29 (22.31)	52 (40.00)	49 (37.69)	
Model 1^*a*^ (95% CI)	1.00 (ref)	1.81 (1.14–2.85)	1.81 (1.15–2.87)	0.06
Model 2^*b*^ HR (95% CI)	1.00 (ref)	1.87 (1.18–2.98)	2.00 (1.20–3.34)	<0.05
Model 3^*c*^ HR (95% CI)	1.00 (ref)	2.00 (1.25-3.22)	2.03 (1.21–3.40)	0.06

*CI, confidence interval; HR, hazard ratio; Ref, reference; T, tertile*.

a *Model 1 unadjusted*.

b *Model 2 adjusted for age at diagnosis and total energy*.

c *Model 3 same as Model 2 and further adjusted for body mass index, comorbidities, diet change, dietary pattern, education, FIGO stage, histological type, histopathologic grade, menopausal status, parity, physical activity, residual lesions, and smoke status*.

d *P-value for linear trend calculated from category median values*.

**Figure 3:**
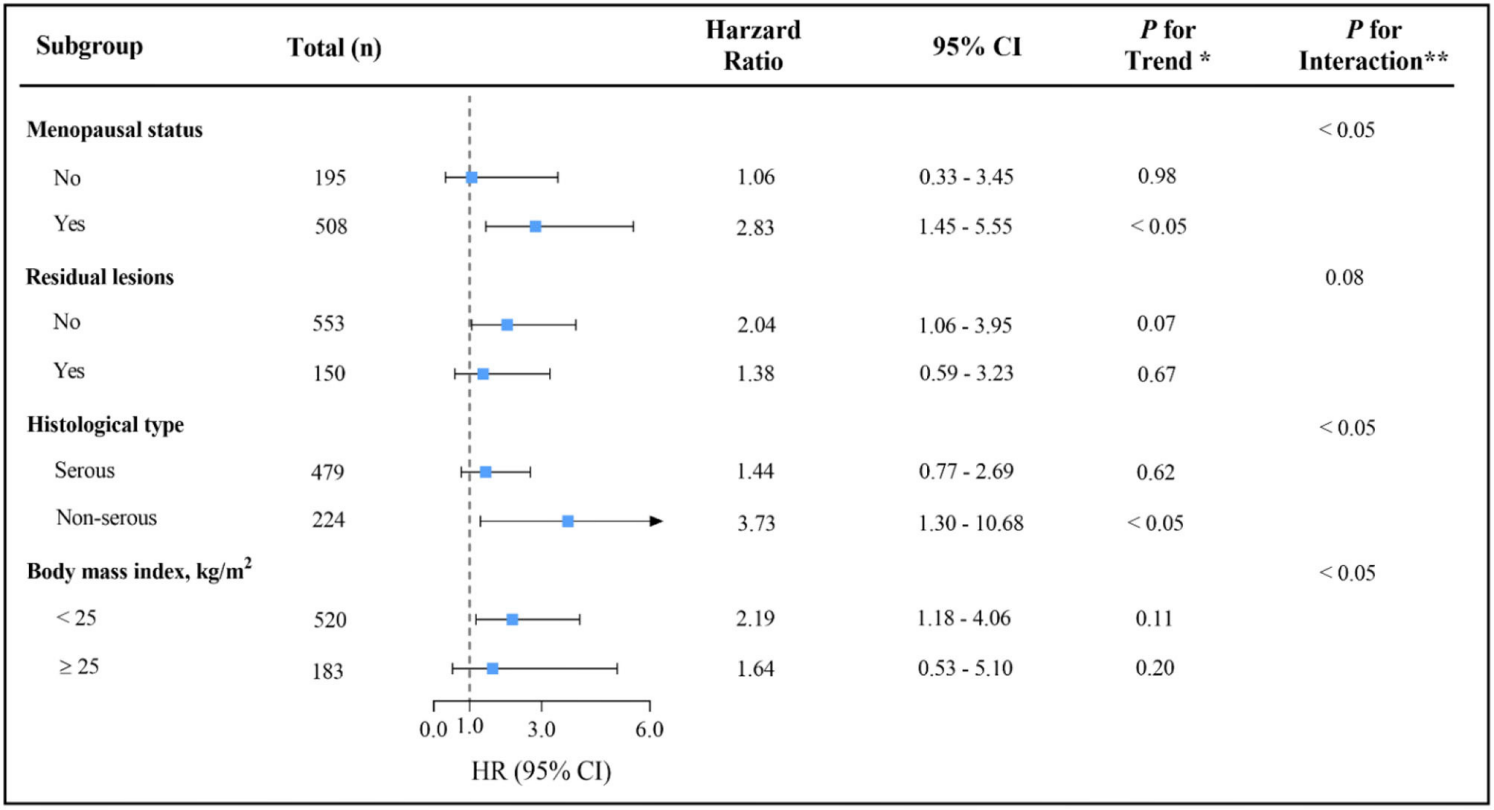
Multivariable hazard ratios (HRs) and 95% CIs for overall survival among ovarian cancer patients across strata of various factors. The analyses used three categories of total dairy intake (T_1_ < 14.76, T_2_ 14.76-90.00 and T_3_ ≥ 90.00g/d). The forest plot represents the HRs of the comparison of the highest versus the lowest of dairy intake. Cox model stratified by menopausal status, residual lesions, histological type and body mass index, with additional adjustments for age at diagnosis, comorbidities, diet change, dietary pattern, education, FIGO stage, histopathologic grade, parity, physical activity, smoke status and total energy.* indicates P for trend across levels of total dairy intake.** indicates P for interaction between strata and total dairy intake.

The influence of total dairy intake on overall survival in OC was examined across potential effect-modifying variables. Of note, the higher mortality risk associated with the highest total dairy intake was present only in menopausal patients, patients with no residual lesions, non-serous patients, or patients with BMI of <25 (Figure 3). Nevertheless, statistical power to adequately examine the differences was limited by the sample size in the above stratified analyses. Such analyses should be considered exploratory. Similar results were obtained for the protein, fat, and calcium from dairy products (Supplementary Figures 4–6). Results among patients with stage III-IV OC were consistent with the main findings, although they were attenuated (data not shown).

## Discussion

In this prospective cohort of 853 women diagnosed with OC, pre-diagnosis dairy product intake was positively associated with total mortality. Similar magnitudes of the mortality increase were observed for pre-diagnosis protein, fat, and calcium from dairy intake.

Findings from prior studies on the association between pre-diagnosis consumption of dairy products and OC survival were limited and inconsistent (Supplementary Table 1). The present findings are in line with a previous longitudinal follow-up study of 341 U.S. women diagnosed with OC, where higher pre-diagnosis intake of all types of milk was associated with worse survival (7). In addition, an earlier cohort study of 609 Australian epithelial OC cases reported a modest relationship between pre-diagnosis dairy intake and poor survival (15). However, results from the three follow-up cohort studies were inconsistent with the present findings (8, 9, 16). Playdon et al. found that pre-diagnosis intake of low-fat or high-fat dairy was not associated with OC survival among 811 Australian women with OC (9). A study conducted by Thomson et al. among 636 U.S. OC patients indicated no correlation between milk consumption and OC survival (8). In Japan, a large and prospective study by Sakauchi et al. followed 64,327 women for an average of 13.3 years, where a total of 77 of them died of OC. This study also showed no association between consumption of milk and dairy products and the survival of OC patients (16). The reason for this inconsistency might be attributable to different ways used to assess the exposure to dairy products. Exposure assessment in study by Playdon et al. was based on the dairy servings. The Thomson et al. study was based on the points of the Healthy Eating Index 2005, while the Sakauchi et al. study was based on the frequency of dairy intake. Exposure assessment in the present study was based on the quantity of dairy intake, which might be more accurate than that in other studies. Moreover, the proportion of advanced stage patients in the present study (III–IV: 48.1%) was obviously smaller than that in the study by Playdon et al. (III–IV: 71.0%). The ethnic composition of the population of the present study (Asian) differed from that of the study by Thomson et al. (mainly white: 88.1%). The study evidence suggested that consumption of dairy products was also different between Chinese and American patients (20). In our study, only 38 (5.4%) OC patients reached Dietary guidelines for Chinese residents recommend intake (300 g/d), and the mean dairy products intake was 84 g/d. This is lower than American patients (20).

Although the current research on the underlying biological mechanisms of dairy product intake and OC prognosis has been scarce, the present study considered the possible effects from the aspects of fat, protein, and calcium content. Dietary fat has been indicated to be related to high levels of circulating estrogen. The present evidence suggests that elevated levels of estrogen may promote growth and proliferation of OC (13). Furthermore, fat and protein dairy product components may be positively implicated in elevating the level of insulin-like growth factor-1 (11, 14). Insulin-like growth factor-1 receptor overexpression can increase the proliferation of OC cells, restrain OC cell apoptosis, or induce malignant transformation of OC cells (10, 12). The receptor-interacting protein kinase 1 might regulate the mitochondrial Ca2+ uptake to promote cancer cell proliferation (21).

The present study had strengths that are worth mentioning. The originality of the work is the principal strength of the present research, because this is the first study to investigate the relationship between pre-diagnostic diary product intake and OC prognosis in China. The prospective and high follow-up rates (93%) reduce the potential for recall and selection bias. A further strength is that the study was rigorously controlled for the majority of potential prognosis-related confounding factors, such as comorbidities, FIGO stage, histological type, and residual lesions. In addition, the potential impact of nutrients, such as fat, calcium, and protein, in dairy products on the prognosis of OC was further explored.

Nevertheless, several limitations exist in the current study. First, since we directly collected the frequency of dairy product intake rather than intake in the questionnaire, the assessment of dairy product intake may be imprecise. However, well-trained investigators as well as validated FFQs were utilized to collect dietary information for OC patients in the study, which might reduce deviation. Second, since the dietary intake of OC patients was obtained using FFQ measurements prior to diagnosis, it may not reflect the intake after diagnosis. However, dairy products constitute a key part of the daily diet. It is possible that the intake of dairy products may not change before and after diagnosis because recent studies have provided limited or weak evidence of the potential effect of dairy products on OC (22). Third, we failed to examine whether associations with OC prognosis and dairy product type differed by subtype, such as skim/low-fat milk, cheese, and yogurt, due to lower intakes of these dairy products in the present study as well as in China (23). In addition, although the impact of pre-diagnostic dairy product intake on progression-free survival of OC patients was not examined, evidence suggested that OC patients might have short post-progression survival because of the high mortality rate and progression-free survival similarity to overall survival (24, 25). Fourth, the pre-diagnosis daily dairy product consumption in the present study (mean: 84.00 g/day) was close to the estimates made by the Shanghai Women's Health Study of 64,191 adult Chinese women (mean: 62.25 g/day) (26). Conversely, the mean intake of dairy products in the U.S. is estimated to be 268.8 g/day (20). Therefore, the present study findings should be interpreted with caution. Fifth, residual confounding factors are a possible concern in any observational study. Although we comprehensively adjusted for the majority of potential confounders to minimize their influence, there was no way to exclude the impact of unknown or unmeasured confounders.

In conclusion, high pre-diagnosis dairy product intake was strongly associated with worse survival in OC patients. This prognostic effect was similar in the analyses of protein, fat, and calcium from dairy. Further studies with longer follow-up periods, as well as analyses of different dairy products, are warranted in the future.

## Data Availability Statement

The raw data supporting the conclusions of this article will be made available by the authors, without undue reservation.

## Ethics Statement

The studies involving human participants were reviewed and approved by Shengjing Hospital of China Medical University. The patients/participants provided their written informed consent to participate in this study.

## Author Contributions

T-TG, X-QL, and Q-JW: study design. SG, X-QL, and SY: collection of data. F-HL and Y-FW: analysis of data. LJ, F-HL, Z-YW, Y-FW, and, RH: drafting the manuscript. LJ, T-TG, F-HL, Z-YW, Y-FW, LJ, and Q-JW: revision of the manuscript. All authors contributed to the article and approved the submitted version.

## Funding

This work was supported by the Natural Science Foundation of China (Nos. 82073647 and 81602918 to Q-JW and No. 82103914 to T-TG), LiaoNing Revitalization Talents Program (No. XLYC1907102 to Q-JW), Shenyang high level innovative talents support program (No. RC190484 to Q-JW), and 345 Talent Project of Shengjing Hospital of China Medical University (No. MJ0268 to Q-JW).

## Conflict of Interest

The authors declare that the research was conducted in the absence of any commercial or financial relationships that could be construed as a potential conflict of interest.

## Publisher's Note

All claims expressed in this article are solely those of the authors and do not necessarily represent those of their affiliated organizations, or those of the publisher, the editors and the reviewers. Any product that may be evaluated in this article, or claim that may be made by its manufacturer, is not guaranteed or endorsed by the publisher.
