# Supplementary material for: Pre-diagnosis Dairy Product Intake and Ovarian Cancer Mortality: Results From the Ovarian Cancer Follow-Up Study (OOPS)
Source: Front Nutr. 2021 Oct 29;8:750801. doi: 10.3389/fnut.2021.750801 (PMC8586538; doi:10.3389/fnut.2021.750801)
Supplement: Supplementary file 1 [file Data_Sheet_1.docx]

Supplementary Table 1 Characteristics of studies examining the association between dairy products and OC survival

| **Authors, reference; year, country** | **Study design** | **Study population (deaths)** | **Dietary assessment** | **Exposure factors** | **Outcome** | **HR (95%CI)** | **Covariates** |
| --- | --- | --- | --- | --- | --- | --- | --- |
| Playdon, 2017, Australia | Cohort study | 811 (547) | FFQ (135 items, past year) | Low-fat dairy  High-fat dairy | Overall mortality | 0.98 (0.73-1.33)  1.11 (0.87-1.42) | Age at diagnosis, International Federation of Gynaecology and Obstetrics stage, amount of residual disease, grade, tumor subtype, smoking status, BMI, physical activity index, marital status, and daily caloric intake. |
| Thomson, 2014, America | Cohort study | 636(354) | FFQ (past year) | Milk | All-cause mortality  Cancer-specific mortality | 1.04 (0.77-1.40)  1.13 (0.82-1.54) | Age at diagnosis, stage at diagnosis, race/ethnicity, diabetes, physical activity, total energy intake, waist circumference, family history of ovarian cancer, and clinical trial arms. |
| Dolecek, 2010, America | Cohort study | 341 (176) | FFQ (60 items, past 3-5year) | Dairy  Dairy (suggested)  Dairy (other)  Milk, all type  Milk, whole  Milk, 2%  Milk, skim or 1%  Dairy excluding milk | Overall survival | 1.51 (0.87-2.61)  1.45 (0.85-2.41)  1.40 (0.81-2.42)  2.15 (1.20-3.84)  1.23 (0.61-2.49)  1.64 (1.00-2.71)  1.07 (0.62-1.83)  0.73 (0.45-1.20) | Age group, race, stage, grade, residual lesions, smoking status, BMI, oral contraceptive use, parity, and total energy intake. |
| Sakauchi, 2007, Japan | Cohort study | 64327 (77) | FFQ (32 items) | Milk  Cheese  Butter  Yogurt | Death | 1.67 (0.66-4.23)  1.66 (0.65-4.25)  1.35 (0.56-3.25)  1.66 (0.71-3.91) | Age, menopausal status, number of pregnancies, history of sex hormone use, BMI, physical activity, and education. |
| Nagle, 2003, Australia | Cohort study | 609 (372) | FFQ (119 items, past year) | Dairy products | Overall survival | 1.30 (0.97-1.74) | Stage, age, grade, total energy intake, BMI. |

FFQ: food frequency questionnaire; BMI: body mass index; HR: hazard ratio


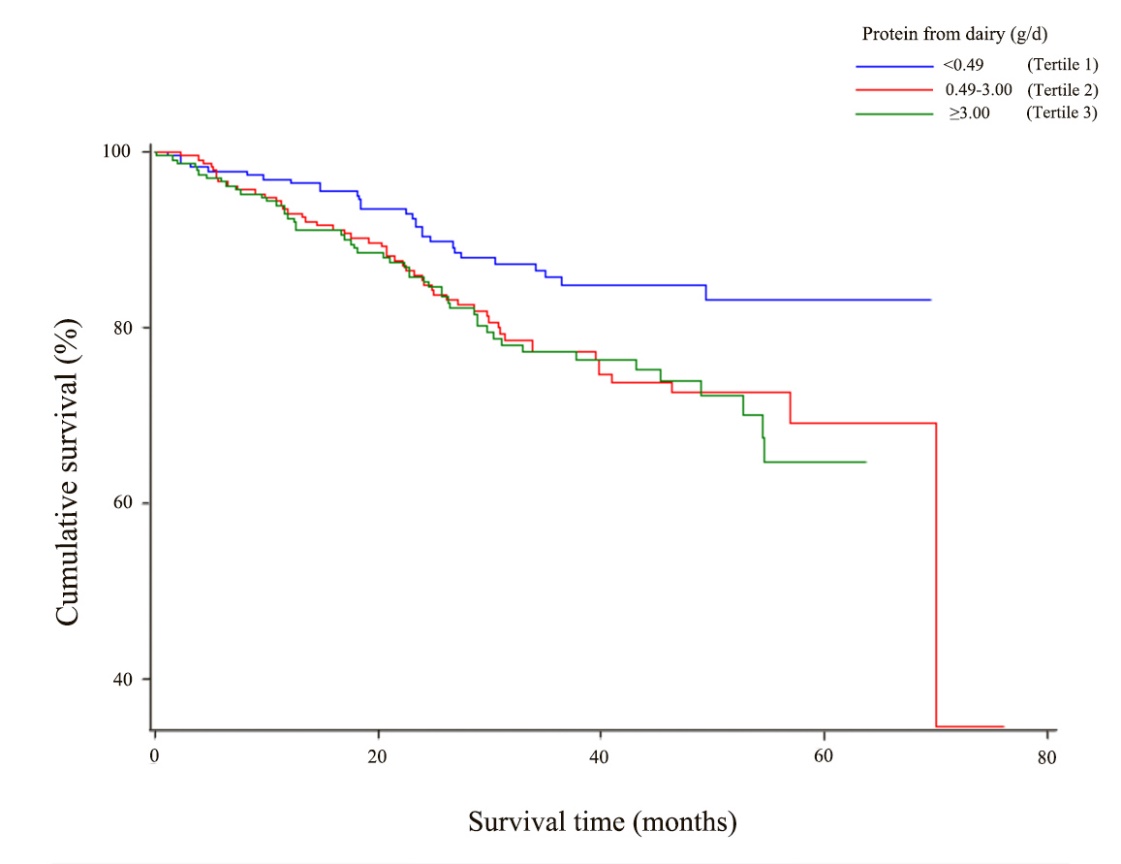


Supplementary Figure 1 Kaplan-Meier survival curves for protein from dairy productions consumption.


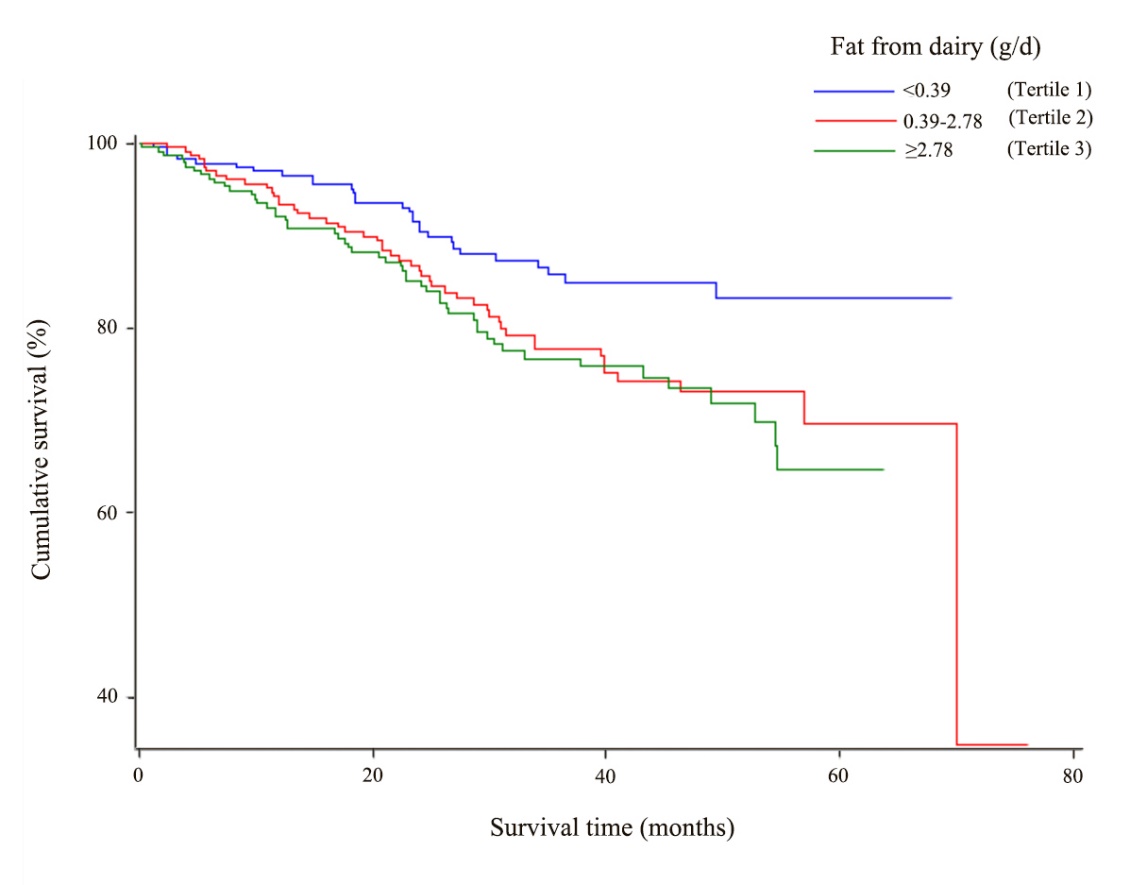


Supplementary Figure 2 Kaplan-Meier survival curves for fat from dairy productions consumption.


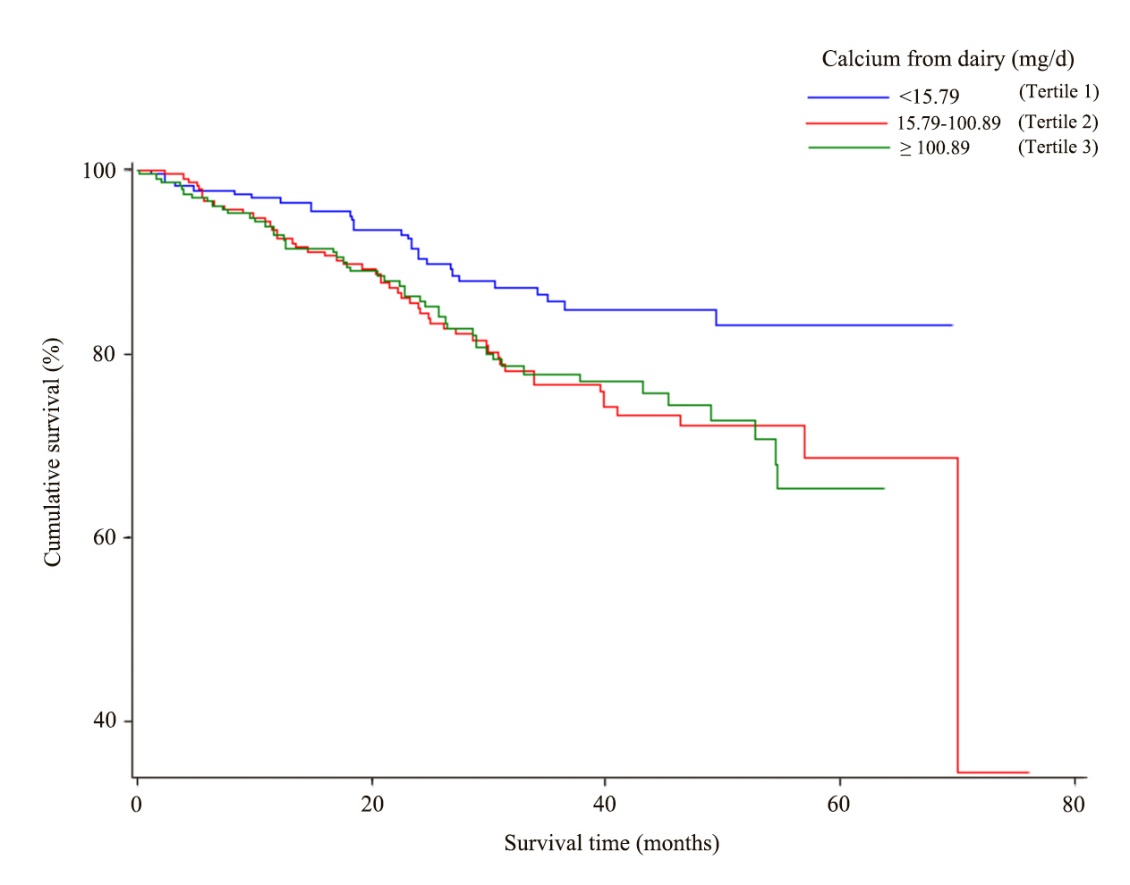


Supplementary Figure 3 Kaplan-Meier survival curves for calcium from dairy productions consumption.


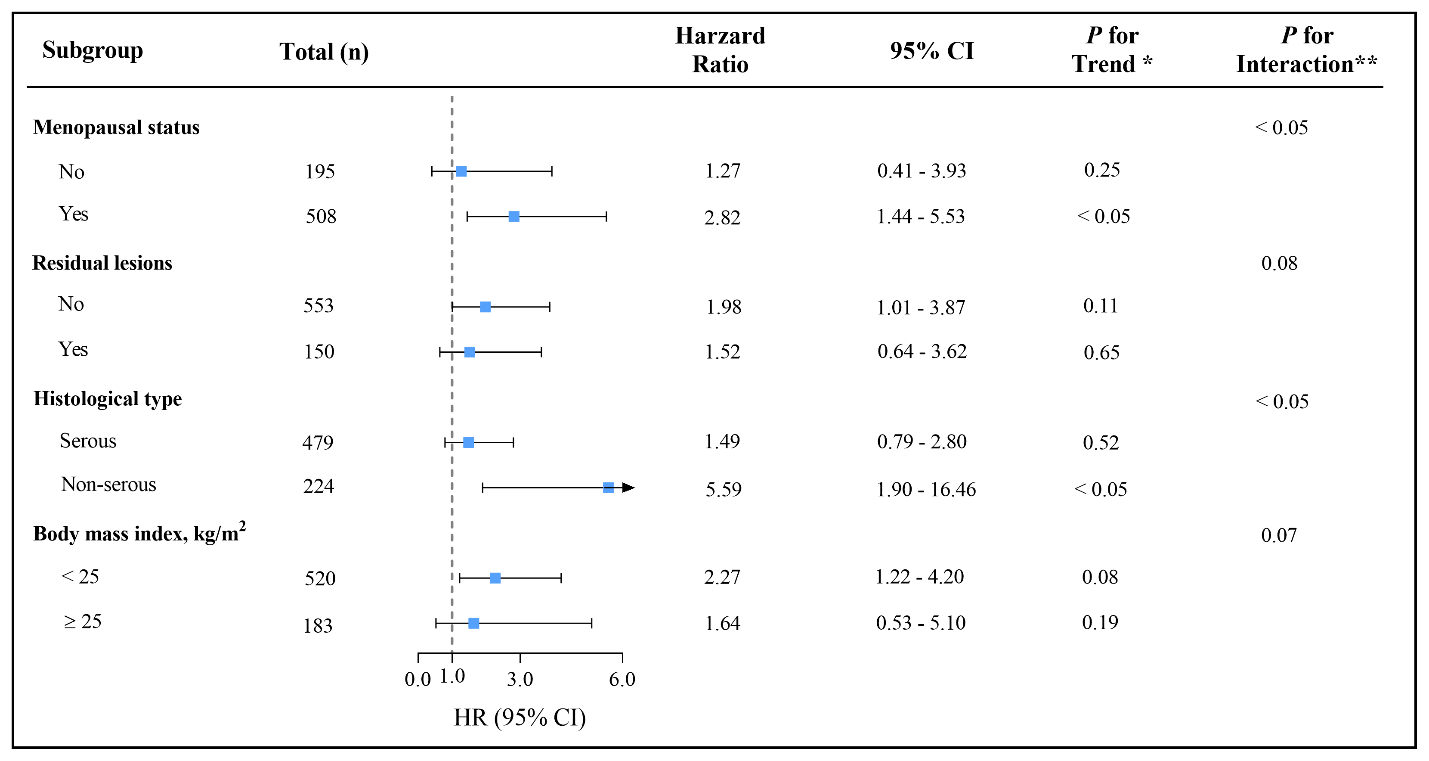


Supplementary Figure 4 Multivariable hazard ratios (HRs) and 95% CIs for overall survival among ovarian cancer patients across strata of various factors. The analyses used three categories of protein from dairy (T_1_＜0.49, T_2_ 0.49-3.00 and T_3_ ≥3.00g/d). The forest plot represents the HRs of the comparison of the highest versus the lowest of dairy intake. Cox model stratified by menopausal status, residual lesions, histological type and body mass index, with additional adjustments for age at diagnosis, comorbidities, diet change, dietary pattern, education, FIGO stage, histopathologic grade, parity, physical activity, smoke status, and total energy intake.

* indicates P for trend across levels of total dairy intake.

** indicates P for interaction between strata and total dairy intake.


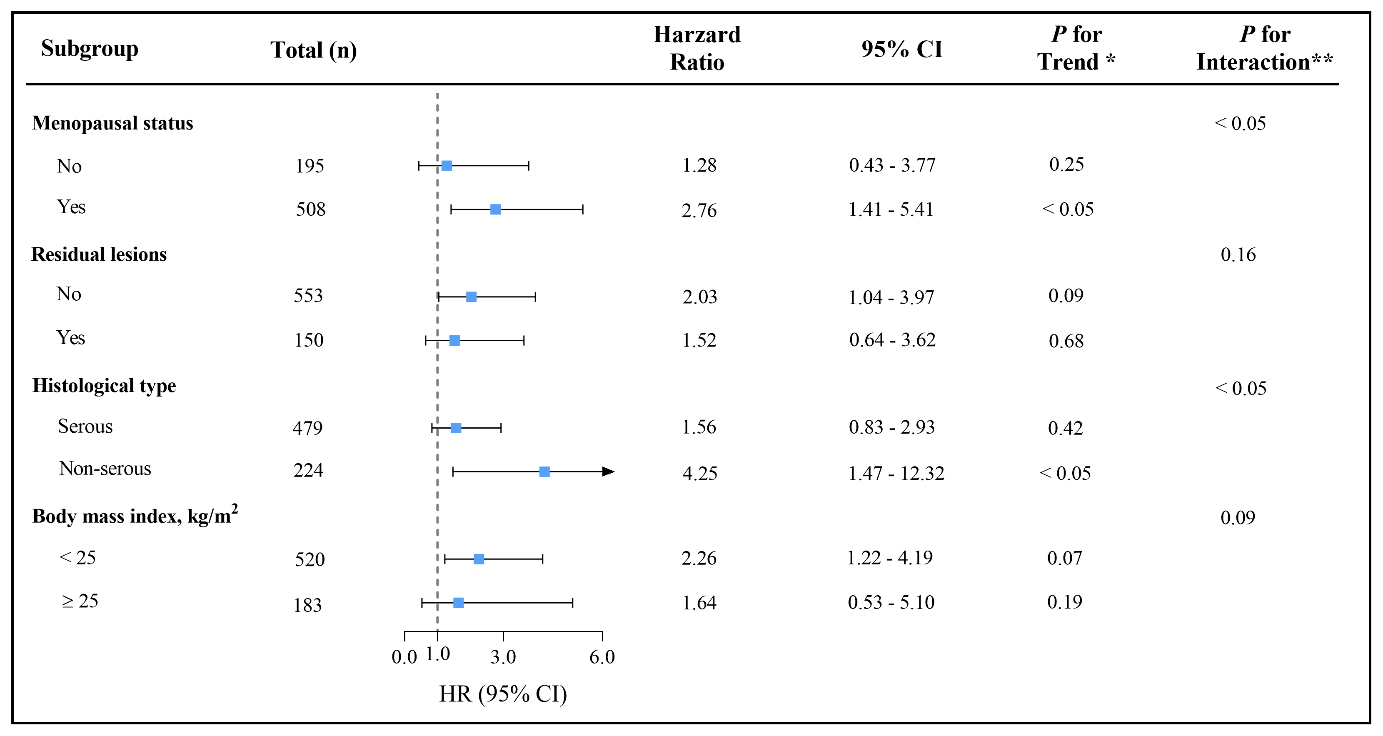


Supplementary Figure 5 Multivariable hazard ratios (HRs) and 95% CIs for overall survival among ovarian cancer patients across strata of various factors. The analyses used three categories of fat from dairy (T_1_＜0.49, T_2_ 0.49-3.00 and T_3_ ≥3.00g/d). The forest plot represents the HRs of the comparison of the highest versus the lowest of dairy intake. Cox model stratified by menopausal status, residual lesions, histological type and body mass index, with additional adjustments for age at diagnosis, comorbidities, diet change, dietary pattern, education, FIGO stage, histopathologic grade, parity, physical activity, smoke status, and total energy intake.

* indicates P for trend across levels of total dairy intake.

** indicates P for interaction between strata and total dairy intake.


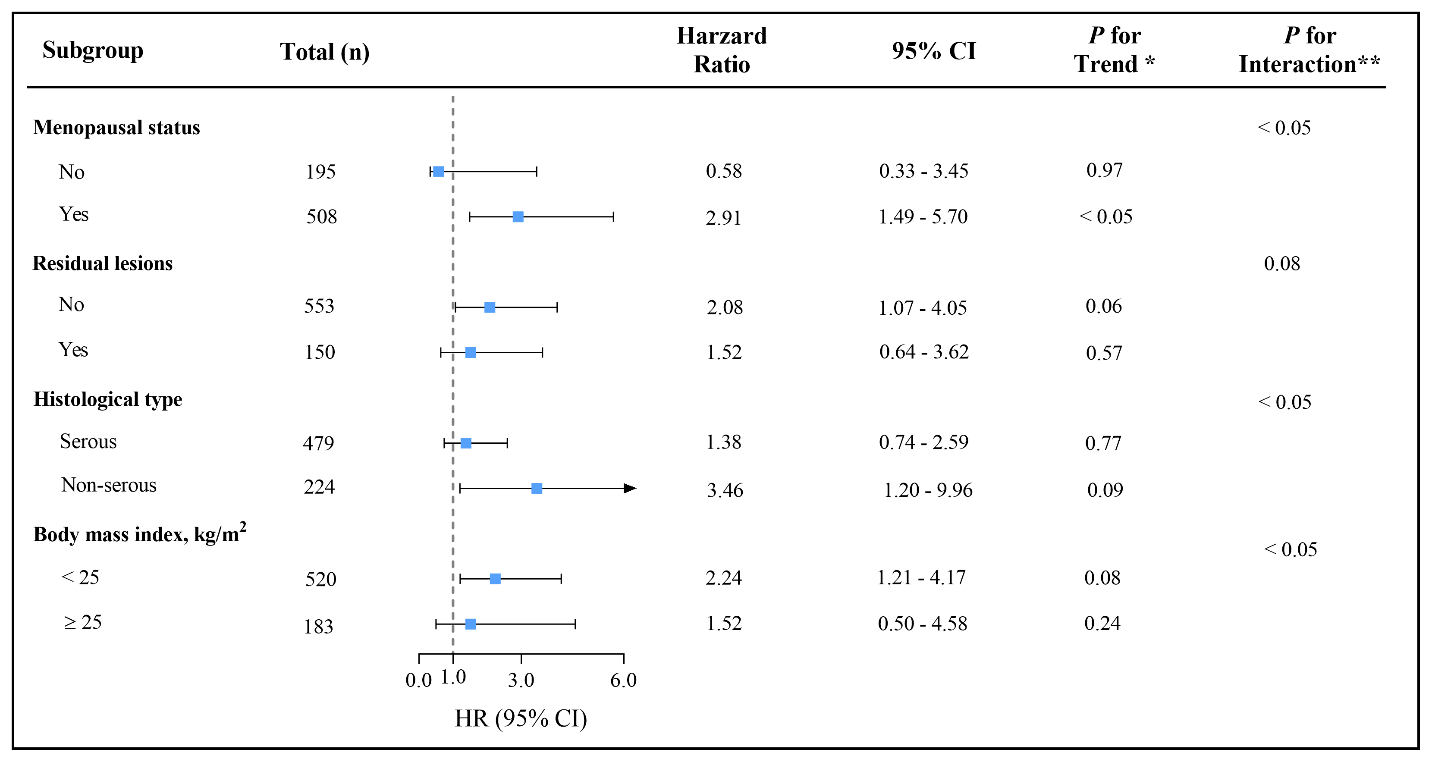


Supplementary Figure 6 Multivariable hazard ratios (HRs) and 95% CIs for overall survival among ovarian cancer patients across strata of various factors. The analyses used three categories of calcium from dairy (T_1_＜15.79, T_2_ 15.79-100.89 and T_3_ ≥100.89mg/d). The forest plot represents the HRs of the comparison of the highest versus the lowest of dairy intake. Cox model stratified by menopausal status, residual lesions, histological type and body mass index, with additional adjustments for age at diagnosis, comorbidities, diet change, dietary pattern, education, FIGO stage, histopathologic grade, parity, physical activity, smoke status, and total energy intake.

* indicates P for trend across levels of total dairy intake.

** indicates P for interaction between strata and total dairy intake.

**Reference:**

Playdon, M.C., Nagle, C.M., Ibiebele, T.I., Ferrucci, L.M., Protani, M.M., Carter, J., Hyde, S.E., Neesham, D., Nicklin, J.L., Mayne, S.T., and Webb, P.M. (2017). Pre-diagnosis diet and survival after a diagnosis of ovarian cancer. *Br J Cancer* 116, 1627-1637. doi: 10.1038/bjc.2017.120.

Thomson, C.A., E, C.T., Wertheim, B.C., Neuhouser, M.L., Li, W., Snetselaar, L.G., Basen-Engquist, K.M., Zhou, Y., and Irwin, M.L. (2014). Diet quality and survival after ovarian cancer: results from the Women's Health Initiative. *J Natl Cancer Inst* 106. doi: 10.1093/jnci/dju314.

Dolecek, T.A., McCarthy, B.J., Joslin, C.E., Peterson, C.E., Kim, S., Freels, S.A., and Davis, F.G. (2010). Prediagnosis food patterns are associated with length of survival from epithelial ovarian cancer. *J Am Diet Assoc* 110, 369-82. doi: 10.1016/j.jada.2009.11.014.

Sakauchi, F., Khan, M.M., Mori, M., Kubo, T., Fujino, Y., Suzuki, S., Tokudome, S., and Tamakoshi, A. (2007). Dietary habits and risk of ovarian cancer death in a large-scale cohort study (JACC study) in Japan. *Nutr. Cancer* 57, 138-45. doi: 10.1080/01635580701274178.

Nagle, C.M., Purdie, D.M., Webb, P.M., Green, A., Harvey, P.W., and Bain, C.J. (2003). Dietary influences on survival after ovarian cancer. *Int. J. Cancer* 106, 264-9. doi: 10.1002/ijc.11204.
